# Supplementary material for: C-Terminal Extended Domain-Independent Telomere Maintenance: Modeling the Function of TIN2 Isoforms in Mus musculus
Source: Int J Mol Sci. 2025 Mar 7;26(6):2414. doi: 10.3390/ijms26062414 (PMC11941968; doi:10.3390/ijms26062414)
Supplement: Supplementary file 1 [file ijms-26-02414-s001.zip › ijms-3406326-supplementary.pdf]

C-Terminal Extended Domain-Independent Telomere Maintenance: Modeling the Function of TIN2 Isoforms in *Mus musculus*

Chiao-Ming Huang [1,2], Yi-Ling Shen [2], Chia-Lo Ho [2], Tzeng-Erh Chen [2], Hsuan-Yu Hsia [2], Zhou Songyang [3] and Liuh-Yow Chen [1,2,*]

[1] Molecular and Cell Biology, Taiwan International Graduate Program, Academia Sinica and Graduate Institute of Life Sciences, National Defense Medical Center, Taipei 11490, Taiwan; iointheair@gate.sinica.edu.tw
[2] Institute of Molecular Biology, Academia Sinica, Taipei 11529, Taiwan; ling0725@gate.sinica.edu.tw (Y.-L.S.); eggcookie@gap.kmu.edu.tw (C.-L.H.); monkichi0802@gmail.com (T.-E.C.); hcu1228@gate.sinica.edu.tw (H.-Y.H.)
[3] Sun Yat-Sen Memorial Hospital, Sun Yat-Sen University, Guangzhou 510275, China; songyang@bcm.edu
* Correspondence: lyowchen@gate.sinica.edu.tw

Supplemental information

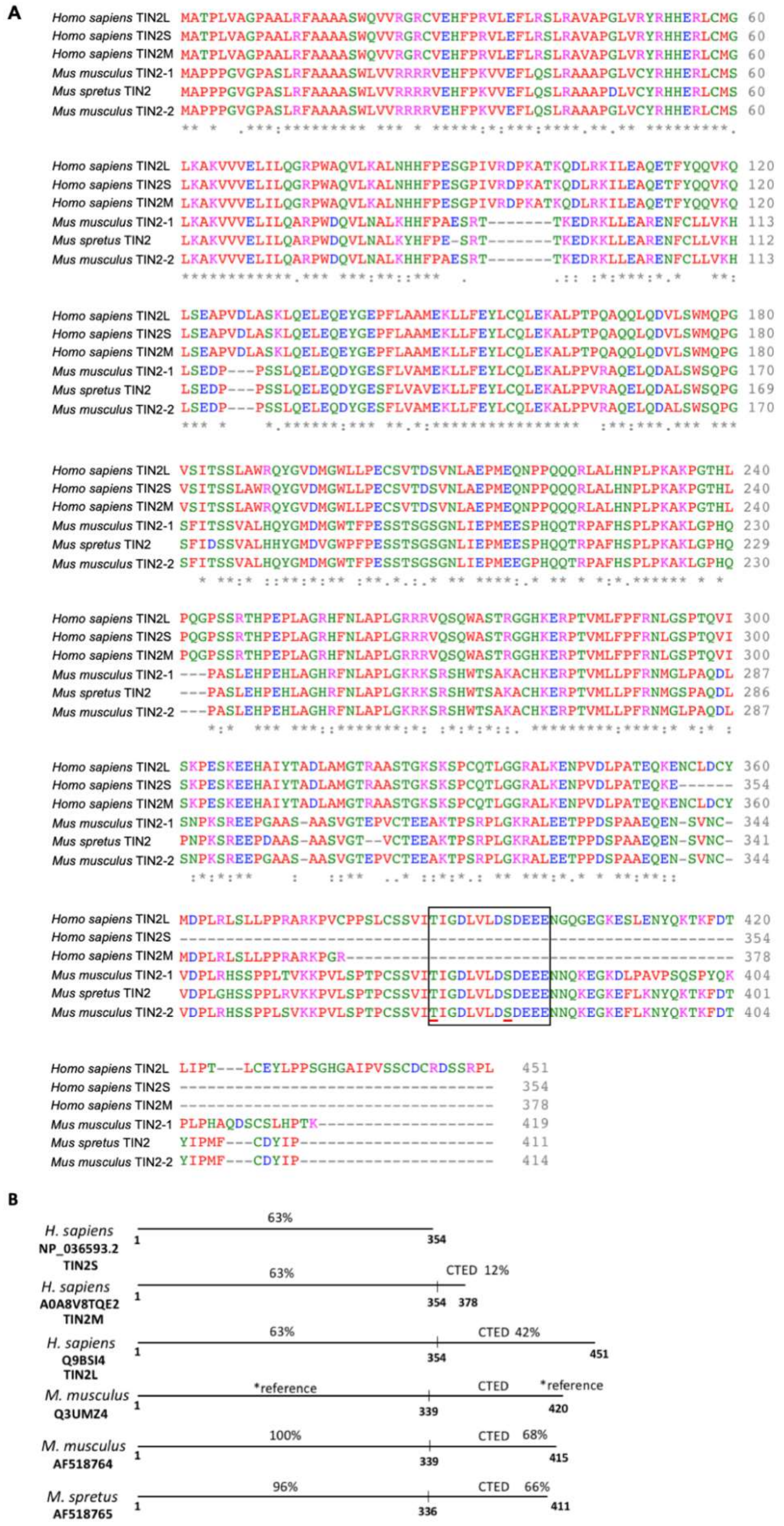

Figure S1. Sequence alignment of human and mouse TIN2. Human TIN2S, TIN2M, TIN2L, 2 distinct *Mus musculus* TIN2, and 1 *Mus spretus* TIN2 sequences were obtained from public databases, including Uniport and NCBI databases. In Homo sapience, NP_0.36593.2 is TIN2S, A0A8V8TQE2 is TIN2M, and Q9BSI4 is TIN2L. In Mus musculus, Q3UMZ4 is mouse TIN2-1, and AF518764/Q8CJ45 is TIN2-2. AF518765 is Mus spretus TIN2 with CTED. (A) Multiple sequence alignment result was obtained with Clustal Omega with default settings for the alignment. (B) Graphical summary for the sequence identity for CTED and N-terminal sequences without CTED in the six protein sequences. The percentage numbers indicate the sequence identity compared to the *M. musculus* sequence Q3UMZ4. The sequence identity was determined in the Needleman-Wunsch algorithm by using the Global Align online tool on the NCBI Blast website. Independent sequences were uploaded separately to the online tool for the analysis. The full-length alignment is provided in Supplementary Table S1.

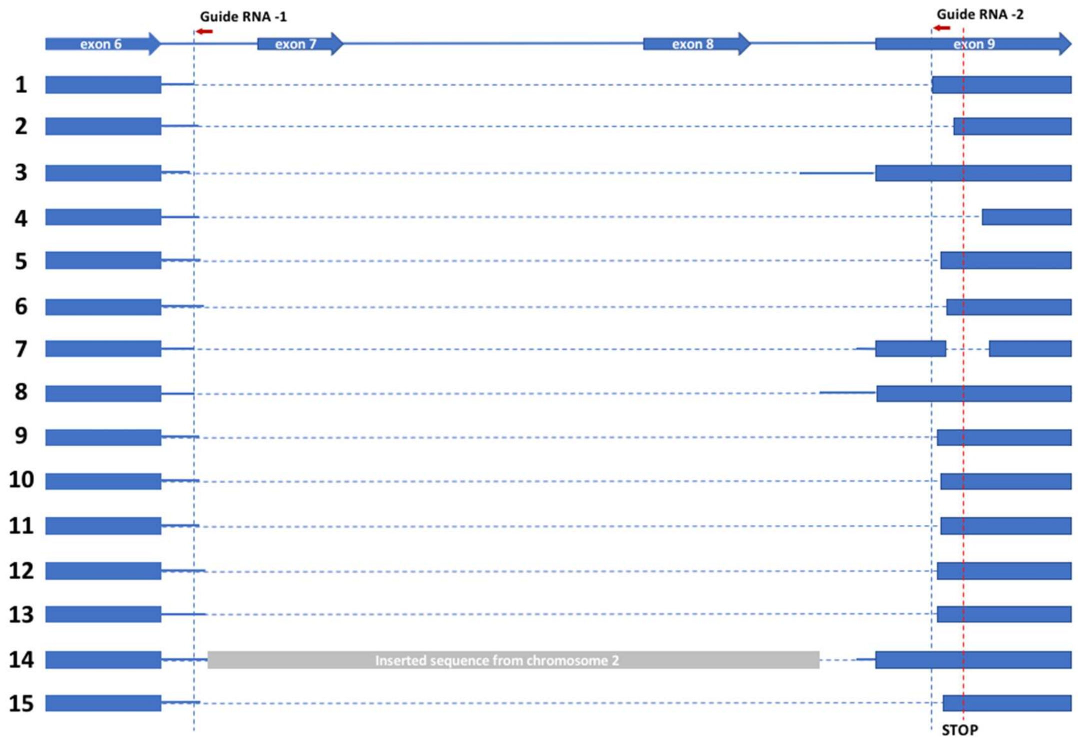

Figure S2. Sequence alignment of modified *Tinf2* LD alleles. Fifteen distinct modified *Tinf2* LD alleles were generated in mice. Genomic DNA was extracted from tail tips and sequenced following PCR amplification. Deleted sequences in the schematic sequence alignment are illustrated by dotted lines.

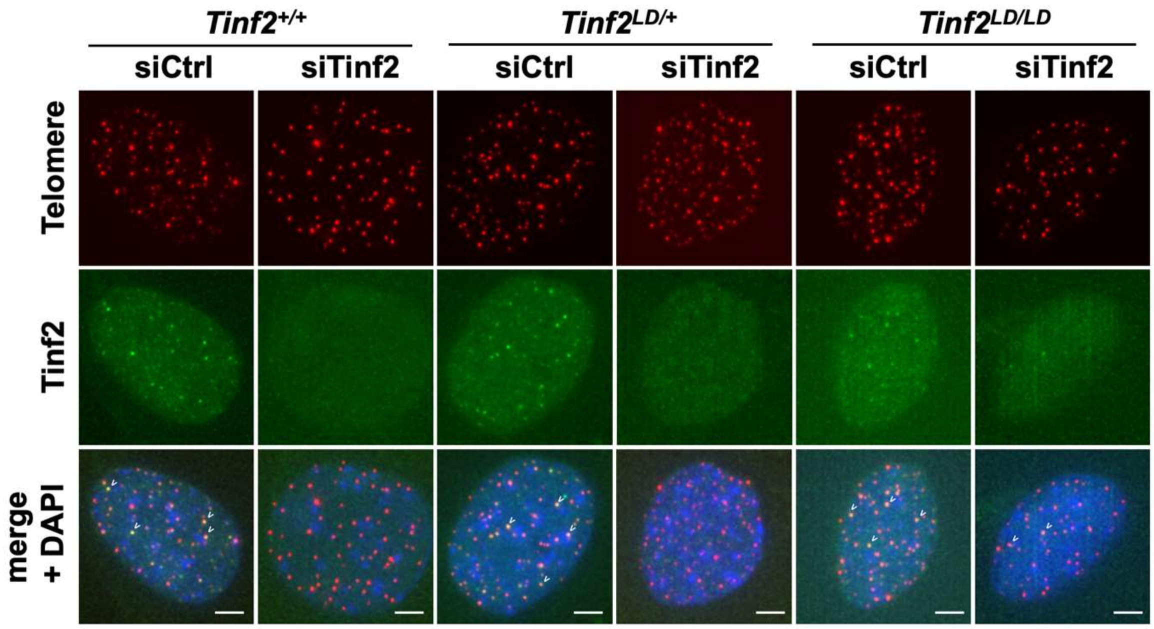

Figure S3. Representative images of IF-FISH illustrating expression of mTINF2 protein and telomere signal in MEFs with siRNA-mediated mTINF2 depletion. Telomere signals were visualized using a telomere probe (red), whereas endogenous mTINF2 signals were detected using mouse anti-Tinf2 antibodies (green). Representative telomere-localized mTINF2 (co-localized signals) is indicated by white arrowheads. Quantitative analysis is shown in Figure 3D. Scale bar: 2 μm.

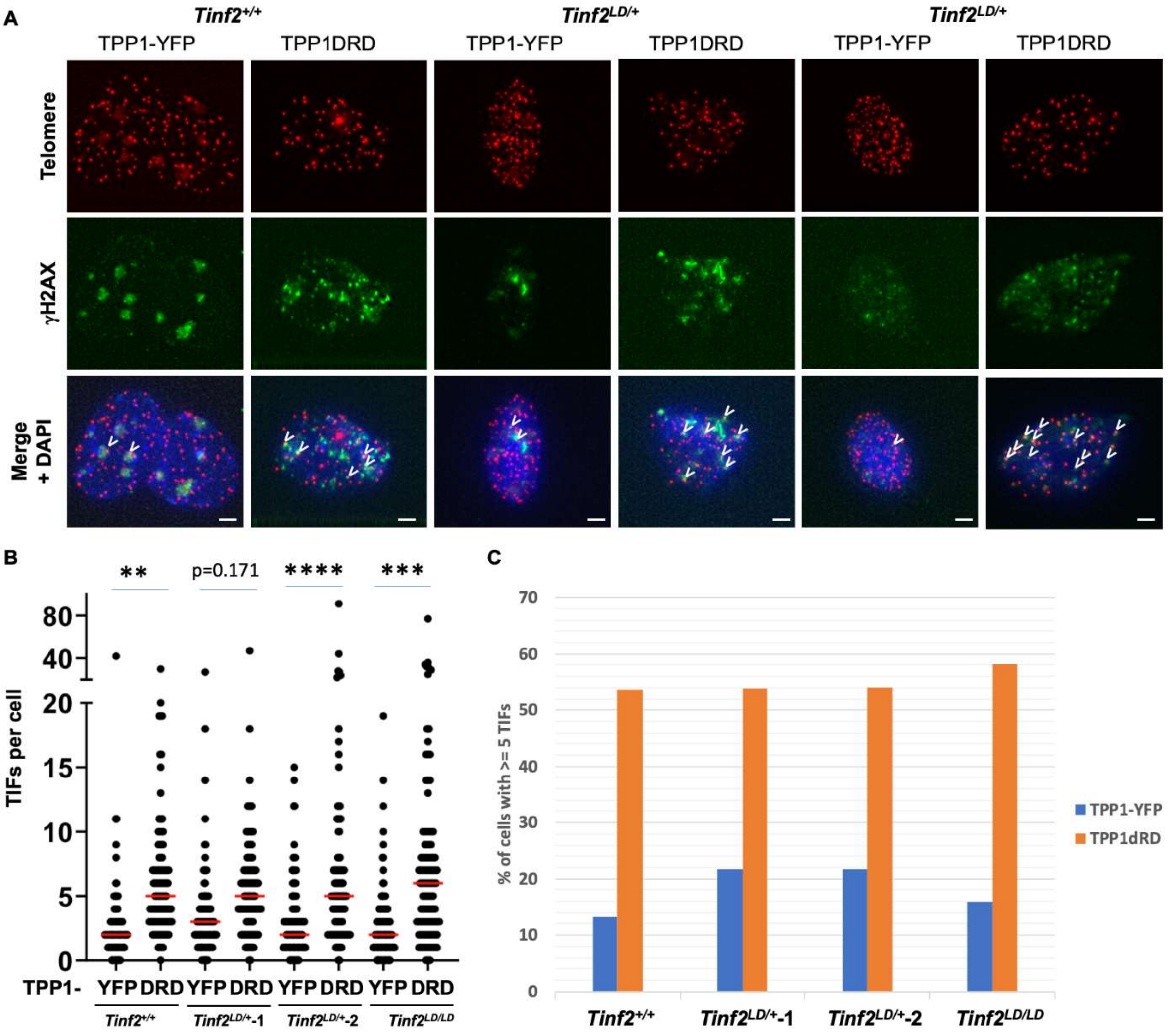

Figure S4. Telomere damage in MEF cells with ectopically expressing TPP1 or dominant-negative TPP1. *Tinf2*$^{LD/+}$ animals were mated to obtain comparable littermates for analysis. Four primary MEF lines were collected from the same litter: one *Tinf2*$^{+/+}$, two *Tinf2*$^{LD/+}$, and one *Tinf2*$^{LD/LD}$. MEF cells were transfected with either a dominant-negative TPP1 construct (TPP1ΔRD) or a control construct (TPP1-YFP) and analyzed for telomere damage response. (A) Representative images of MEF cells expressing the ectopic constructs. Phosphorylated H2AX (γH2AX) signals are shown in green, and telomere signals are visualized using a telomere probe in red. White arrows indicate colocalized γH2AX and telomere puncta, marking telomere damage-induced foci (TIFs). Cells with more than five TIFs were classified as TIF+ cells. (B) Quantification TIFs per cell. Data represent over 100 cells assessed in each group. Statistical

analysis was performed using one-way ANOVA. **$p < 0.01$, ***$p < 0.001$, ****$p < 0.0001$. In *Tinf2*$^{+/+}$, the mean difference between siCtrl and siTinf2 is -3.439 (95% confident intervals (CI): -6.189 to -0.6886; Cohen's d: 0.591). In *Tinf2*$^{LD/+}$-1, the mean difference is -2.28 (95% CI: -4.982 to 0.4227; Cohen's d: 0.418). In *Tinf2*$^{LD/+}$-2, the mean difference is -5.278 (95% CI: -7.980 to -2.575; Cohen's d: 0.709). In *Tinf2*$^{LD/LD}$, the mean difference is -4.101 (95% CI: -6.817 to -1.386; Cohen's d: 0.591). (C) Percentages of TIF+ cells across different MEF lines. Scale bar: 2 μm.

A

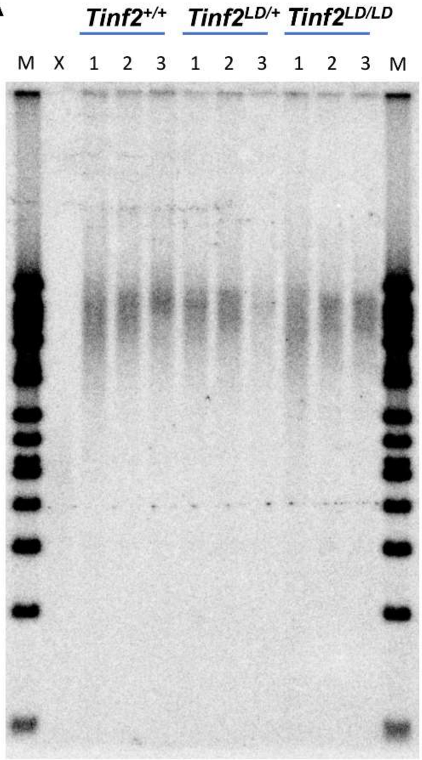

Tinf2+/+
Tinf2LD/+
Tinf2LD/LD
M X 1 2 3 1 2 3 1 2 3 M

B

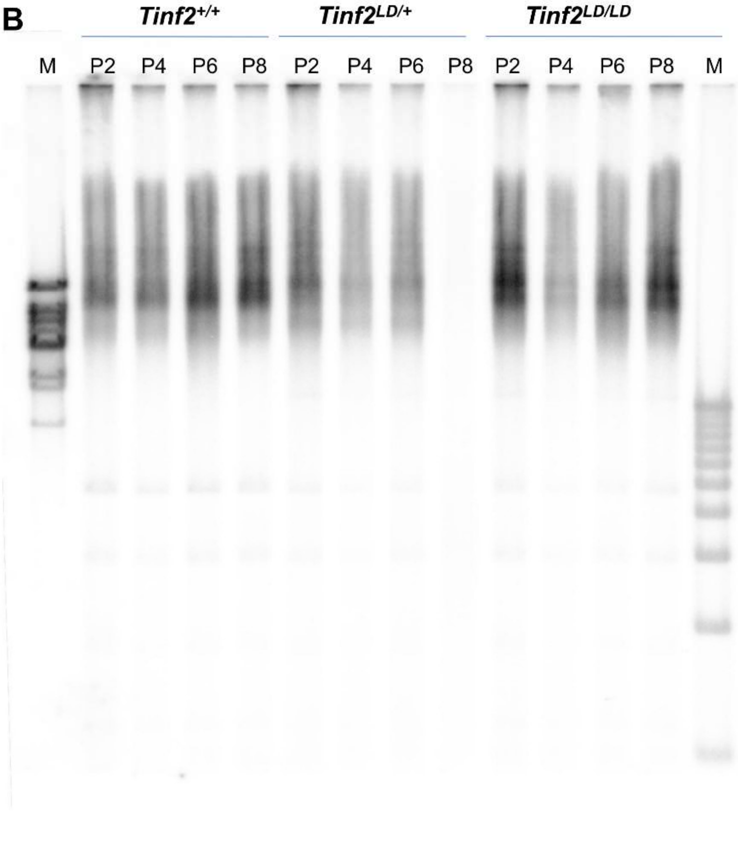

Tinf2+/+
Tinf2LD/+
Tinf2LD/LD
M P2 P4 P6 P8 P2 P4 P6 P8 P2 P4 P6 P8 M

Figure S5. Telomere restriction fragment (TRF) analysis in *Tinf2*$^{+/+}$, *Tinf2*$^{LD/+}$, and *Tinf2*$^{LD/LD}$ MEFs. (A) TRF assay results from three independent *Tinf2*$^{+/+}$, *Tinf2*$^{LD/+}$, and *Tinf2*$^{LD/LD}$ MEF lines. Genomic DNA was extracted from early-passage primary MEF lines (passage 2-4), digested with RsaI and HinfI restriction enzymes, and separated by agarose gel electrophoresis to assess telomere length polymorphism. Southern blot hybridization with a (TTAGGG) telomeric probe was used to visualize telomere repeats. (B) TRF assay results for *Tinf2*$^{+/+}$, *Tinf2*$^{LD/+}$, and *Tinf2*$^{LD/LD}$ MEF lines across multiple passages (passage 2-8). M: molecular weight marker; X: unrelated samples; P: passage number.

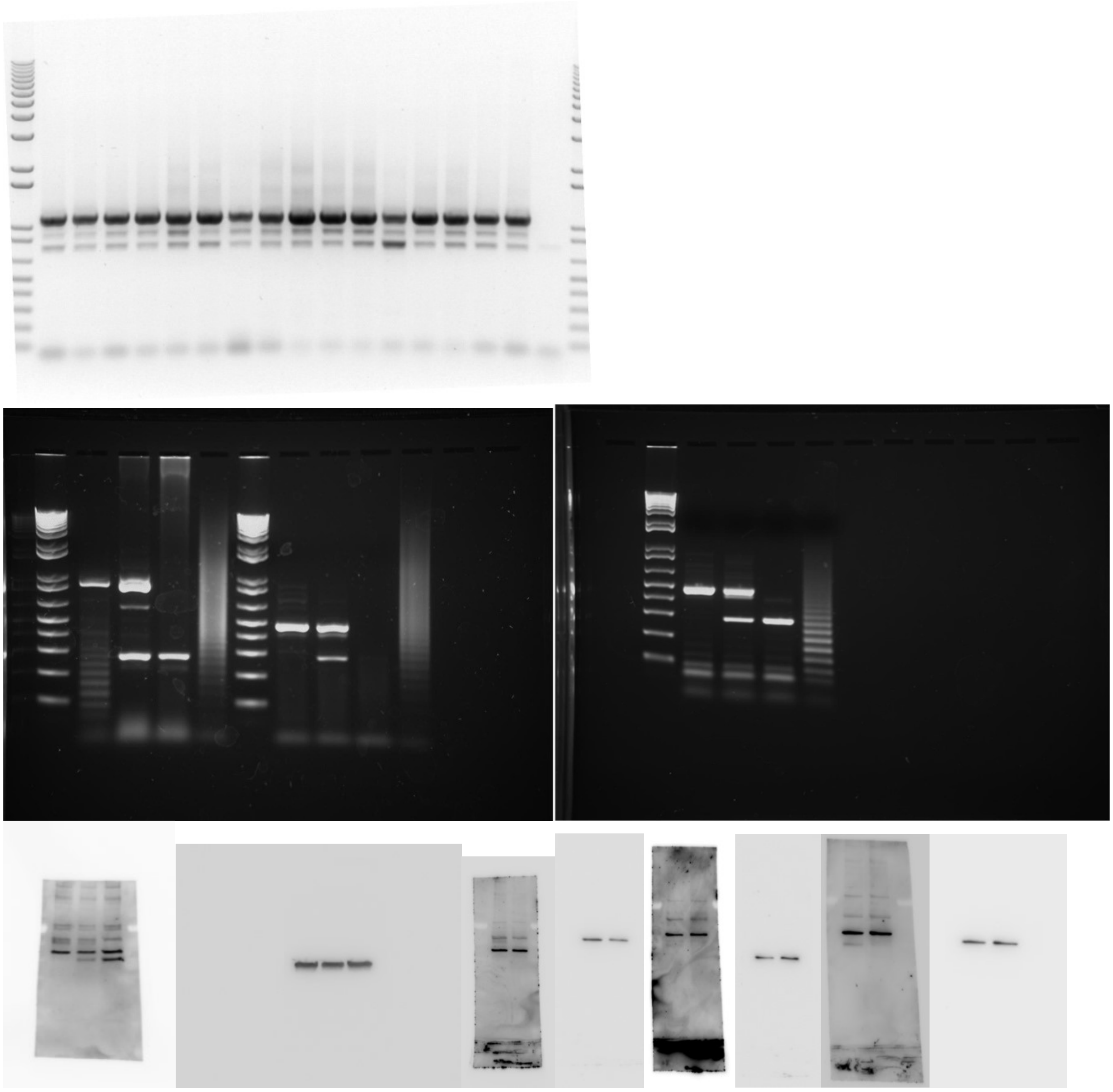

Figure S6. Full-length gels and blots.

Species / ID		Homo sapiens			Mus musculus		Mus spretus
		TIN2S (NP_096593.2)	TIN2M (Uniport_ A0A8V8TQW2)	TIN2L (Uniport Q9BSI4)	Uniport Q3UMZ4	AF518764	AF518765
Homo sapiens	TIN2S (NP_096593.2)	--	--	--	51%	52%	50%
	TIN2M (Uniport_ A0A8V8TQW2)	--	--	--	53%	54%	53%
	TIN2L (Uniport Q9BSI4)	--	--	--	54%	61%	59%
Mus musculus	Uniport Q3UMZ4	51%	53%	58%	--	94%	90%
	AF518764	52%	54%	61%	94%	--	90%
Mus spretus	AF518765	50%	53%	59%	90%	90%	--

Supplementary Table S1. Full protein sequence alignment results. Human TIN2S, TIN2M, TIN2L, 2 distinct *Mus musculus* TIN2, and 1 *Mus spretus* TIN2 sequences were obtained from public databases, including Uniport and NCBI databases. The percentage numbers indicate the sequence identity for two compared sequences. Sequence identity was determined in the Needleman-Wunsch algorithm by using the Global Align online tool on the NCBI Blast website.

Figure 3D				
Group 1	Group 2	Mean difference	95% CI	Effect size (Cohen's d)
Tinf2$^{+/+}$, siCtrl	*Tinf2*$^{+/+}$, siTinf2	0.4795	0.4216 to 0.5374	2.17
Tinf2$^{LD/+}$, siCtrl	*Tinf2*$^{LD/+}$, siTinf2	0.3905	0.3329 to 0.4481	1.81
Tinf2$^{LD/LD}$, siCtrl	*Tinf2*$^{LD/LD}$, siTinf2	0.3163	0.2567 to 0.3758	1.47
Figure 3E				
Group 1	Group 2	Mean difference	95% CI	Effect size (Cohen's d)
Tinf2$^{+/+}$, siCtrl	*Tinf2*$^{+/+}$, siTinf2	-2.063	-2.739 to 1.387	0.483
Tinf2$^{LD/+}$, siCtrl	*Tinf2*$^{LD/+}$, siTinf2	-2.085	-2.762 to -1.407	0.487
Tinf2$^{LD/LD}$, siCtrl	*Tinf2*$^{LD/LD}$, siTinf2	-1.839	-2.538 to -1.180	0.430
Tinf2$^{+/+}$, siCtrl	*Tinf2*$^{LD/+}$, siCtrl	-0.1549	-0.8301 to 0.5204	0.036
Tinf2$^{LD/+}$, siCtrl	*Tinf2*$^{LD/LD}$, siCtrl	-0.1958	-0.8760 to 0.4844	0.046
Tinf2$^{+/+}$, siCtrl	*Tinf2*$^{LD/LD}$, siCtrl	-0.3507	-1.028 to 0.3268	0.083
Figure 3F				
Group 1	Group 2	Mean difference	95% CI	Effect size (Cohen's d)
Tinf2$^{+/+}$, siCtrl	*Tinf2*$^{+/+}$, siTinf2	-23	-31.25 to -14.75	3.16
Tinf2$^{LD/+}$, siCtrl	*Tinf2*$^{LD/+}$, siTinf2	-24.67	-32.92 to -16.41	3.39
Tinf2$^{LD/LD}$, siCtrl	*Tinf2*$^{LD/LD}$, siTinf2	-24.33	-32.59 to -16.08	3.34
Tinf2$^{+/+}$, siCtrl	*Tinf2*$^{LD/+}$, siCtrl	0	-8.253 to 8.253	0
Tinf2$^{LD/+}$, siCtrl	*Tinf2*$^{LD/LD}$, siCtrl	-1.667	-9.920 to 6.586	0.23
Tinf2$^{+/+}$, siCtrl	*Tinf2*$^{LD/LD}$, siCtrl	-1.667	-9.920 to 6.586	0.23

Supplementary Table S2. Statistical information for Figure 3

Genotype	*Tinf2*$^{+/+}$	*Tinf2*$^{LD/+}$	*Tinf2*$^{LD/LD}$
Male	33 (18.3%)<br>Predicted: 12.5%	42 (23.3%)<br>Predicted: 25%	19 (10.6%)<br>Predicted: 12.5%
Female	22 (12.2%)<br>Predicted: 12.5%	44 (24.4%)<br>Predicted: 25%	20 (11.1%)<br>Predicted: 12.5%

Supplementary Table S3. Summary of offspring from *Tinf2*$^{LD/+}$ matings. Summary of the genotypes of pups generated from mating male *Tinf2*$^{LD/+}$ and female *Tinf2*$^{LD/+}$ mice. A total of 180 offspring were recorded. Predicted outcomes are based on Mendelian inheritance. Chi-square test of Hardy-Weinberg equilibrium determined the chi-square value in male is 0.697, in female is 0.049, and in all gender is 0.244.

Parent genotype	*Tinf2*$^{LD/LD}$								
Parent generation	G1	G1	G1	G2	G2	G3	G4	G5	G6
Male	3	4	7	4	4	5	2	4	3
Female	1	2	1	3	2	5	4	1	2
Total	4	6	8	7	6	10	6	5	5
Parent genotype	*Tinf2*$^{+/+}$								
Parent generation	G1	G2	G3	G4	G5	G6			
Male	4	3	4	4	4	N/A			
Female	5	4	4	2	2	N/A			
Total	9	7	8	6	6	5			

Supplementary Table S4. Summary of offspring from matings in different generation of *Tinf2*$^{+/+}$ and *Tinf2*$^{LD/LD}$ animals. Record of pups generated from indicated mating. N/A: no information with genders.
